# Rumination, Hopelessness, Behavioural Avoidance and Psychopathology Symptoms After Bereavement: Serial Mediation Analyses

**DOI:** 10.1002/cpp.70053

**Published:** 2025-03-10

**Authors:** Maarten C. Eisma, Antje Janshen, Nienke de Haan

**Affiliations:** ^1^ University of Groningen Groningen the Netherlands

**Keywords:** bereavement, complicated grief, coping, emotion regulation, perseverative cognition, repetitive negative thought

## Abstract

Bereavement can precipitate severe mental health problems, including major depressive disorder and prolonged grief disorder. Rumination is a risk factor of post‐loss mental health problems, and as such, a better understanding of its working mechanisms may inform clinical practice. Rumination is theorized to take up time and increase feelings of hopelessness, leading to inactivity and social withdrawal, which in turn fuels post‐loss psychopathology. Yet, these ideas have not been tested comprehensively. Therefore, we aimed to fill this gap in knowledge. A sample of bereaved adults (87% women) completed questionnaires on socio‐demographic and loss‐related characteristics, rumination, hopelessness, behavioural avoidance of activities, and depressive and prolonged grief symptoms. Two serial mediation analyses demonstrated that rumination may have both direct effects and indirect effects via hopelessness and behavioural avoidance on depressive and prolonged grief symptom levels. Sensitivity analyses, including reverse mediation analyses, supported the validity of the results. Findings show that hopelessness and behavioural avoidance may act as working mechanisms in the relationship between rumination and post‐loss psychopathology. Therapies targeting hopelessness and social withdrawal, such as problem‐solving training and behavioural activation, may be helpful in reducing rumination and depressive and prolonged grief symptoms in bereaved persons.

**Summary**

- Rumination is a risk factor for post‐loss mental health problems.
- Hopelessness and behavioural avoidance of activities mediated the relationship between rumination and prolonged grief symptoms.
- Hopelessness and behavioural avoidance of activities mediated the relationship between rumination and depressive symptoms.
- Therapeutic techniques aimed at increasing hope and activity levels may help reduce rumination and improve post‐bereavement mental health.

Rumination, the process of thinking repetitively and/or recurrently about the causes and consequences of negative events and negative emotions, is recognized as a trans‐diagnostic risk factor for affective and stress‐related disorders (e.g., Aldao, Nolen‐Hoeksema, and Schweizer 2010; Ehring and Watkins 2008; McEvoy et al. 2013). Rumination can be successfully changed through psychotherapy (e.g., Querstret and Cropley 2013; Spinhoven et al. 2018). Psychotherapies targeting rumination have been shown to be successful in the treatment of depression, anxiety, posttraumatic stress and prolonged grief symptoms (e.g., Eisma, Boelen, et al. 2015; Mak et al. 2024; van der Heiden, Muris, and Molen 2012; Watkins et al. 2011; Wenn et al. 2019). The effects of such interventions may be enhanced by improving our understanding of how rumination affects mental health in different populations and applying such knowledge in clinical practice. In the present study, we therefore aimed to conduct a test of one of the proposed working mechanisms of rumination on mental health in a bereaved adult sample.

Although many contemporary theories on rumination exist, the Response Style Theory (RST) has historically received most scientific attention. In brief, the RST holds that rumination leads to increases in distress by (a) increasing the accessibility and salience of negative cognitions, (b) interfering with problem‐solving, (c) impeding instrumental behaviour and (d) driving away social support (Nolen‐Hoeksema 2001). In a revision of the RST, a fifth mechanism, behavioural avoidance, was added (Nolen‐Hoeksema, Wisco, and Lyubomirsky 2008). This mechanism entails that rumination helps people avoid an aversive environment by occupying attention and time and by building a case that one is facing a hopelessly uncontrollable situation that is impossible to change. Rumination thus not only removes people from aversive situations but also provides them with reasons to become inactive and withdraw from activities. Decreased participation in social, occupational and recreational activities reduces access to experiences that counter negative cognitions about the self, life and the future, which fuels negative affect and increases depressive and prolonged grief symptoms (Martell, Addis, and Jacobson 2001; Boelen, van den Hout, and van den Bout 2006). Specifically in bereaved adults, inactivity and social withdrawal may also limit gaining new experiences in which the absence of the deceased is strongly felt, which could interfere with the cognitive processing of the loss (Boelen, van den Hout, and van den Bout 2006).

In line with these notions, rumination is concurrently and longitudinally related to post‐loss mental health, including depressive symptoms and prolonged grief symptoms (e.g., Eisma et al. 2013; Eisma, Schut, et al. 2015; Smith, Wild, and Ehlers 2024; Thimm et al. 2024; for reviews: Eisma and Stroebe 2017, 2021). Moreover, behavioural avoidance mediated the effects of rumination on depressive symptoms in a cross‐sectional survey (Eisma, de Lang, and Boelen 2020) and a longitudinal survey of bereaved adults (Eisma et al. 2013). Additionally, behavioural avoidance only mediated the effects of rumination on prolonged grief symptoms concurrently (Eisma, de Lang, and Boelen 2020), but not longitudinally (Eisma et al. 2013). The literature in the bereavement field thereby broadly aligns with studies demonstrating positive associations of rumination with behavioural avoidance and mental health problems in nonbereaved samples (e.g., Moulds et al. 2007; for reviews: Aldao, Nolen‐Hoeksema, and Schweizer 2010; Naragon‐Gainey, McMahon, and Chacko 2017). Further indirect evidence for the role of behavioural avoidance as a mechanism explaining the relationship between rumination and mental health problems comes from randomized controlled trials demonstrating the effectiveness of behavioural activation in reducing rumination and mental health problems in bereaved and nonbereaved samples (e.g., Eisma, Boelen, et al. 2015; Papa et al. 2013; McIndoo et al. 2016; Moshier and Otto 2017). Based on the RST, behavioural activation likely achieves such effects because increasing the number of valued activities reduces feelings of hopelessness and limits the time available for ruminative coping.

While these theoretical notions derived from the RST thus generally align with findings from empirical research, the putative role of hopelessness in the relationship between rumination and post‐loss mental health has not yet been investigated. This seems a notable omission, because if hopelessness partly explains the downward spiral of rumination, behavioural inactivity and post‐loss mental health problems in bereaved adults, reducing it may be a viable way to break this spiral. In nonbereaved samples, multiple interventions have been found to effectively reduce hopelessness, such as problem‐solving therapy, which focuses on training problem‐solving skills (for a review: Townsend et al. 2001). If hopelessness is a working mechanism in the relationship between rumination and post‐loss mental health, such interventions may prove helpful for bereaved populations. An additional reason to clarify the associations of hopelessness, coping and mental health following bereavement is that hopelessness is considered a risk factor of suicidal ideation and behaviours (Abramson, Metalsky, and Alloy 1989; Liu et al. 2015), which is a serious and harmful consequence for a minority of bereaved persons (e.g., Sekowski and Prigerson 2022; Fisher et al. 2024).

Based on these theoretical and empirical notions, the present study sought to clarify the associations between rumination, hopelessness, behavioural avoidance and depressive and prolonged grief symptomatology in bereaved adults. Following the RST (Nolen‐Hoeksema, Wisco, and Lyubomirsky 2008), we hypothesized that bereaved adults who ruminate more would experience more hopelessness, which relates to reduced participation in social, occupational and recreational activities, which, in turn, relates to higher depressive and prolonged grief symptom levels. We aimed to conduct a preliminary test of these hypotheses by conducting two serial mediation analyses based on data from a cross‐sectional survey among bereaved adults.

## 1 Methods

### 1.1 Procedure and Participants

The present study's cross‐sectional data was collected within the framework of a three‐wave longitudinal survey study, wherein the impact of post‐loss rumination on mental health was examined (Eisma, Schut, et al. 2015). Ethical approval for the procedures in this study was obtained from the Ethics Committee for Psychology of Utrecht University. People were eligible to participate if they were adults (≥ 18 years old) who had experienced the death of a family member at least 6 months ago. Prospective participants were recruited through advertisements displayed on bereavement organization websites and via Google Ads. All advertisements provided a hyperlink to a dedicated research website where people could access an information page (including information on, e.g., voluntariness, advantages and disadvantages of participation and data handling). After providing online informed consent, participants could complete an online questionnaire programmed in NetQuestionnaires.

At the end of the online survey, participants were asked whether they would be interested to fill in additional surveys 6 and 12 months after filling in the first questionnaire. The present cross‐sectional study utilizes data from participants at the third wave. Prior research showed no significant differences in socio‐demographic or loss‐related characteristics and symptom levels between those who did and did not complete all survey waves (Eisma, Schut, et al. 2015). Table 1 shows a summary of the socio‐demographic and loss‐related characteristics of the sample at baseline.

**TABLE 1:** Socio‐demographic and loss‐related sample characteristics (*N* = 242).

Socio‐demographic variables	
Sex, *n* (valid %)	
Man	31 (13)
Woman	210 (87)
Age in years, *M* (SD)	48.72 (11.71)
Highest education level, *n* (valid %)	
Lower education (other than college, university)	149 (62)
Higher education (college, university)	91 (38)
Loss‐related variables	
Sex of the deceased, *n* (valid %)	
Man	150 (62)
Woman	92 (38)
Relationship with the deceased, *n* (valid %)	
Partner	123 (51)
Child	22 (9)
Parent	22 (9)
Sibling	73 (30)
Cause of death, *n* (valid %)	
Nonviolent (natural causes)	213 (89)
Violent (i.e., accident, homicide, suicide)	27 (11)
Death was, *N* (valid %)	
Expected	105 (43)
Unexpected	110 (45)
Both or neither	27 (11)
Time in months since death, *M (SD)*	9.62 (8.32)

## 2 Materials

### 2.1 Socio‐demographic and Loss‐Related Characteristics

Socio‐demographic characteristics of participants (i.e., age in years, sex and educational level) and loss‐related characteristics of the deceased (i.e., kinship, time since loss in months, cause of death, and expectedness of the death) were measured using a self‐constructed questionnaire.

### 2.2 Rumination

The brooding subscale of the Ruminative Response Scale (RRS) (Treynor, Gonzalez, and Nolen‐Hoeksema 2003; Dutch version: Schoofs, Hermans, and Raes 2010) was utilized to measure maladaptive depressive rumination. This subscale comprises five items, where participants are asked to indicate, on a four‐point scale ranging from ‘almost never’ (1) to ‘almost always’ (4), how often they engage in certain behaviours when in a negative mood. An example item is ‘I think about a recent event, wishing it had gone better’. Schoofs, Hermans, and Raes (2010) examined the reliability and validity of the Dutch version of the RRS. The subscale demonstrated an acceptable internal consistency for the brooding subscale of the RRS in their study, *α* = 0.78. In the current sample, a similar internal consistency for the brooding subscale was observed, *α* = 0.79.

### 2.3 Hopelessness

Beck's Hopelessness Scale (BHS) (Beck et al. 1974; Dutch version: Stas et al. 2021) was utilized to measure hopelessness. The BHS is a 20‐item self‐report questionnaire. The BHS measures three facets of hopelessness: feelings about the future (e.g., ‘I look forward to the future with hope and enthusiasm’ ‐ negatively formulated), future expectations (e.g., ‘I don't expect to get what I really want’) and loss of motivation (e.g., ‘I might as well give up because I can't make things better for myself’). Participants rate whether the statements are ‘true’ (1) or ‘false’ (0). Stas et al. (2021) reported a good reliability of the BHS in a Dutch clinical sample, *α* = 0.89. In the current sample, the BHS showed excellent reliability, *α* = 0.90.

### 2.4 Behavioural Avoidance

The Depressive and Anxious Avoidance of Prolonged Grief Questionnaire (DAAPGQ) (Boelen and van den Bout 2010) was utilized to measure avoidance behaviour. The DAAPGQ assesses two mechanisms linked to prolonged grief: ‘anxious avoidance’ and ‘depressive avoidance’. We used the five items measuring depressive avoidance, which measure behavioural avoidance of activities. A sample item is: ‘Since ___ is dead, I do much less of the things that I used to enjoy’. In their validation paper, Boelen and van den Bout (2010) showed that the depressive avoidance subscale of the DAAPGQ showed excellent reliability, *α* = 0.90, which was also found in the current sample, *α* = 0.92.

### 2.5 Prolonged Grief Symptoms

The Inventory of Complicated Grief Revised (ICG‐R) (Prigerson and Jacobs 2001; Dutch version: Boelen et al. 2003) was utilized to measure prolonged grief symptoms. The Dutch version of the ICG‐R comprises 29 statements reflecting prolonged grief symptoms. Participants were asked to indicate the extent to which they experienced these symptoms in the past month on a five‐point scale, ranging from ‘almost never’ (0) to ‘always’ (4). An example item is: ‘I feel numb since he/she passed away’. Boelen et al. (2003) investigated the reliability and validity of the Dutch version of the ICG‐R and reported excellent internal consistency for the instrument, *α* = 0.94. The reliability of the ICG‐R was also excellent in the present sample, *α* = 0.95.

### 2.6 Depressive Symptoms

The Hospital Anxiety and Depression Scale (HADS) (Zigmond and Snaith 1983; Dutch version: Spinhoven et al. 1997) was utilized to measure depressive symptoms. The HADS comprises two subscales (depression and anxiety), each comprising seven items. In the present study, we used the depression subscale to measure depressive symptoms. Participants were asked to rate the applicability of statements to them on a four‐point scale (ranging from 0 to 3) regarding their feelings over the past four weeks. An example item from the depression subscale is: ‘I feel as if I am slowed down’. Spinhoven et al. (1997) examined the reliability of the HADS across various Dutch participant groups (both clinical and non‐clinical), revealing acceptable to good reliability for the depression subscale in the different participant groups, *α*'s range = 0.71–0.86. In the current sample, the HADS depression subscale showed excellent reliability, *α* = 0.91.

## 3 Statistical Analyses

All analyses were conducted using R software (R Core Team 2021). Bivariate relationships among the main study variables (rumination, hopelessness, behavioural avoidance, prolonged grief and depressive symptoms) were explored, along with standard measures of location and spread, as a preliminary analysis. Next, using multiple linear regressions, the relationships between the socio‐demographic and loss‐related variables and prolonged grief and depressive symptoms were assessed. Variables that significantly related to prolonged grief or depression symptoms were included as covariates in the main analyses.

Subsequently, a missing data analysis was conducted to inspect if there were differences between completers (i.e., participants who had no missing data on any of the measures, *N* = 111) and noncompleters (i.e., participants who had missing data on at least one of the measures, *N* = 131) on the main variables and covariates. To ensure that the assumption of multivariate normality would hold for our main analysis, Chained Equations (MICE) in R (van Buuren 2018) handled missing data using Multivariate Imputation. MICE generates multiple complete datasets by replacing missing values with estimates based on a selected imputation method. Each imputed dataset is analysed individually, and results are combined following Rubin's rules to account for the number of imputations and any additional variance introduced by the imputation process (van Buuren 2018). A total of 50 imputations produced 50 complete datasets (*N* = 242). Thus, the final sample included 242 observations on all variables. Imputation models were tailored to the variables' characteristics: continuous variables were imputed using predictive mean matching (van Buuren 2018). The models included education level, cause of death, gender of the deceased, relationship to the deceased, time since the loss and the main study variables (rumination, hopelessness, behavioural avoidance, prolonged grief and depressive symptoms). These variables were selected because they predicted missingness and/or were related to the main variables. Multicollinearity among predictors was assessed using the Variance Inflation Factor (*VIF*), with no *VIF* values exceeding the traditional threshold of 10 (range: 1.33–3.21). A comparison of the Means and SDs of the main study variables of the sample with missing data and the sample with imputed data are presented in Table 2.

**TABLE 2:** Comparison of descriptive statistics between the sample with missing data and the sample with imputed data.

	Sample with missing data				Sample with imputed data			
Variables	*M*	Min	Max	*N*	*M*	Min	Max	*N*
Rumination	9.26	4.00	18.00	153	9.34	4.00	18.00	242
Hopelessness	6.74	0.00	19.00	129	6.67	0.00	19.00	242
Behavioural avoidance	16.36	5.00	40.00	152	16.49	5.00	40.00	242
Prolonged grief symptoms	40.18	0.00	93.00	141	41.64	0.00	93.00	242
Depression symptoms	6.68	0.00	18.00	154	6.63	0.00	18.00	242

The main analyses consisted of two serial mediation analyses conducted using structural equation modelling (SEM). The analysis was carried out using the ‘lavaan’ package. The models were estimated using maximum likelihood estimation with robust (Huber‐White) standard errors (i.e., MLR) and model fit was evaluated following Kline's minimum set of reported fit statistics (i.e., 𝜒^2^, comparative fit index (CFI), root‐mean‐square error of approximation (RMSEA) with a 90% CI, and standardized root mean square residual (SRMR) Kline 2016). For CFI, values > 0.90 represent acceptable fit (and values > 0.95 representing excellent fit), and for the RMSEA and the SRMR values < 0.10 represent acceptable fit (and values < 0.05 excellent fit). The size of the standardized beta coefficients was interpreted using Cohen's guidelines for effect sizes of correlations (small effect: *r*/*β* = 0.10–0.29, moderate effect: *r*/*β* = 0.30–0.49, large effect: *r*/*β* ≥ 0.50; cf. Cohen 1988).

**FIGURE 1:**
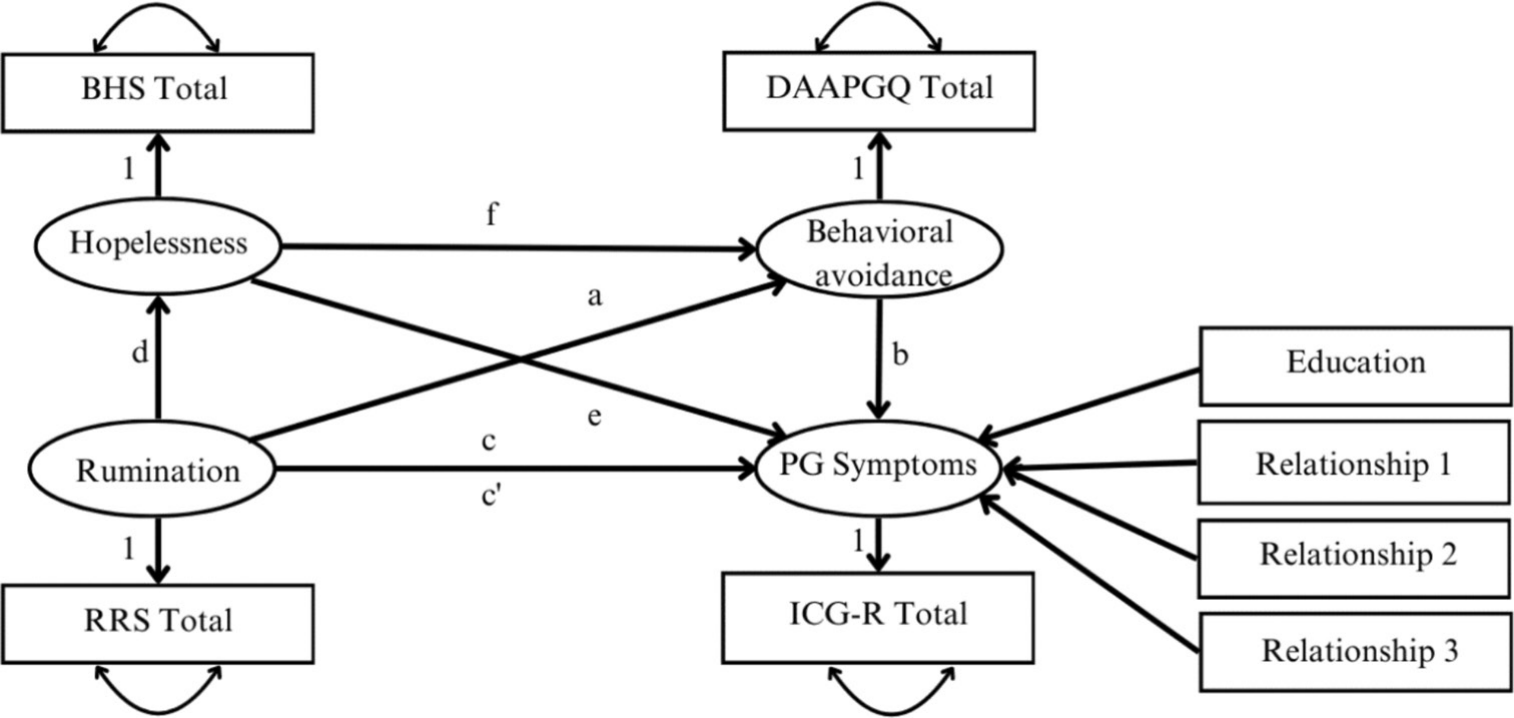
Planned serial mediation analyses predicting prolonged grief symptoms. *Note:* Depression symptoms were the dependent variable in the second serial mediation. BHS Total = Beck's Hopelessness Scale; RRS Total = brooding subscale of the Ruminative Response Scale; DAAPGQ Total = depressive avoidance subscale of the Depressive and Anxious Avoidance of Prolonged Grief Questionnaire; ICG‐R Total = Inventory of Complicated Grief Revised; PG Symptoms = prolonged grief symptoms. The following variables were dummy‐coded: Education = lower education (0) vs. higher education (1); Relationship 1 = partner (1) vs. parent (0); Relationship 2 = child (1) vs. parent (0); Kinship 3 = sibling (1) vs. parent (0).

In the first model, rumination was considered the independent variable, hopelessness and behavioural avoidance were serial mediators, and prolonged grief symptoms were the dependent variable (see Figure 1). The second serial mediation model contained the same variables but utilized depression symptoms as the dependent variable. Multiple paths were specified within each serial mediation model. The total effect (c‐path) indicated the effect of the independent variable (i.e., rumination) on the dependent variable (i.e., prolonged grief or depression symptoms) while controlling for the covariates. The path from rumination to the dependent variable whilst the mediators were also controlled for, represented the direct effect (c′‐path). Additionally, three indirect effects were estimated. First, the indirect effect of rumination via behavioural avoidance (path a) on the dependent variable (path b). Second, the indirect effect of rumination via hopelessness (path d) on the dependent variable (path e), and third the indirect effect of rumination via hopelessness (path d) and behavioural avoidance (f) on the dependent variable (b). The measurement error of the single indicators was fixed (1‐reliability*variance of variable) as proposed by Bollen (1989) to avoid assuming perfect measurement. Furthermore, to test whether there is a significant difference between the indirect effects, contrasts (i.e., indirect effect 1 – indirect effect 2; indirect effect 1 – indirect 3; indirect effect 2 – indirect effect 3) were included in the model. A significant result indicates that there are significant differences between the indirect effects.

We conducted multiple sensitivity checks. First, a 𝜒^2^‐difference test was conducted to inspect whether the more complex models (i.e., including indirect effects via hopelessness and behavioural avoidance) fit the data better than the simplest models (i.e., only including the direct effect from rumination to the dependent variables). Second, another 𝜒^2^‐difference test was conducted to compare the complex models against models in which the paths to and from hopelessness were constrained to zero. A significant 𝜒^2^‐difference test was interpreted to indicate that the more complex model fit the data better. Lastly, as a check of the validity of the model, two reverse serial mediation models were estimated (i.e., prolonged grief or depressive symptoms as independent variables, rumination as the dependent variable).

## 4 Results

### 4.1 Data Inspection

One hundred eleven participants had no missing data on any of the variables included in the models. Analysis of the missing data showed no significant differences between participants who completed the study and those who did not on rumination, behavioural avoidance, prolonged grief symptoms, or the covariates. However, individuals with missing data showed significantly higher levels of hopelessness, *t*(22.82) = −2.33, *p* = 0.029, 95% CI [−5.82, −0.35] and depression symptoms, *t*(75.94) = −2.23, *p* = 0.029, 95% CI [−3.71, −0.21].

Bivariate correlations are shown in Table 3. There were moderate positive associations between rumination and hopelessness, rumination and behavioural avoidance and rumination and depression symptoms. There were strong associations between the other main variables.

**TABLE 3:** Zero‐order correlations between main variables.

	(1)	(2)	(3)	(4)
(1) Rumination	—			
(2) Hopelessness	0.46	—		
(3) Behavioural avoidance	0.49	0.66	—	
(4) Prolonged grief symptoms	0.56	0.67	0.71	—
(5) Depression symptoms	0.48	0.77	0.74	0.78

*Note:* All correlations are significant (*p* < 0.05).

Next, the relationship between background variables and the main variables was inspected. The first model which included prolonged grief symptoms as the dependent variable was significant, *F*(11,119) = 2.79, *p* = 0.002. The background variables explained 20% of the variance in T1 prolonged grief symptoms. Higher education (vs. lower education) (*β* = −0.19, *p* = 0.036) predicted lower prolonged grief symptoms while partner loss (vs. losing another family member, reference category: parental loss; *β* = 0.25, *p* = 0.035) predicted higher levels of prolonged grief symptoms. The second model which included depression symptoms as the dependent variable was significant, *F*(11, 131) = 3.52, *p* < 0.001. The background variables explained 23% of the variance in depression symptoms. Higher education (vs. lower education; *β* = −0.22, *p* = 0.013) predicted lower depression symptoms while partner (*β* = 0.33, *p* = 0.003) and child loss (*β* = 0.20, *p* = 0.024; vs. losing another family member, reference category: parental loss) predicted higher levels of depression symptoms. Thus, education level and relationship with the deceased were included as covariates in the main analysis.

### 4.2 Main Analyses

#### 4.2.1 Full Serial Mediation Model Predicting Prolonged Grief Symptoms

The serial mediation model predicting prolonged grief symptoms showed acceptable fit (𝜒^2^ = 297.627, *df* = 22, *p* = 0.000; *CFI* = 0.922; *RMSEA* = 0.087, *CI* [0.053; 0.122]; *SRMR* = 0.085), explaining 73% of the variance in prolonged grief symptoms (*R* ^2^ = 0.73).

The direct effect (c‐path; other paths were fixed to zero) of rumination on prolonged grief symptoms including covariates was significant (*β* = 0.69, *p* < 0.001) indicating that rumination has a strong positive effect on prolonged grief symptoms. Once all other theorized pathways were estimated, the direct effect (c′‐path) became smaller (i.e., moderate effect) but remained significant, *β* = 0.41, *p* < 0.001.

**FIGURE 2:**
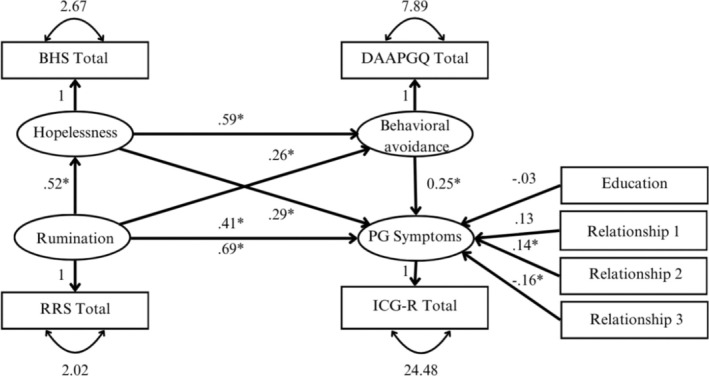
Full serial mediation model predicting prolonged grief symptoms. *Note:* Standardized regression coefficients are depicted. For clarity, the variances of the covariates and the residual errors were omitted. BHS Total = Beck's Hopelessness Scale; RRS Total = brooding subscale of the Ruminative Response Scale; DAAPGQ Total = depressive avoidance subscale of the Depressive and Anxious Avoidance of Prolonged Grief Questionnaire; ICG‐R Total = Inventory of Complicated Grief Revised; PG = prolonged grief. The following variables were dummy‐coded: Education = lower education (0) vs. higher education (1); Relationship 1 = partner (1) vs. parent (0); Relationship 2 = child (1) vs. parent (0); Kinship 3 = sibling (1) vs. parent (0).

Indirect effects of rumination via hopelessness on prolonged grief symptoms (*β* = 0.06, *p* = 0.043), of rumination via behavioural avoidance on prolonged grief symptoms (*β* = 0.15, *p* = 0.007) and via hopelessness and behavioural avoidance on prolonged grief symptoms (*β* = 0.08, *p* = 0.023), were small, positive and significant. The contrast comparing the indirect effect via hopelessness and the indirect effect via behavioural avoidance was not significant (*β* = −0.09, *p* = 0.235). The contrast comparing the indirect effect via hopelessness and the indirect effect via hopelessness and behavioural avoidance was also not significant (*β* = −0.01, *p* = 0.605). The contrast between the indirect effect via behavioural avoidance and the indirect effect via hopelessness and behavioural avoidance was also not significant (*β* = 0.08, *p* = 0.316). These findings indicate that there are no significant differences between the indirect effects. Figure 2 illustrates the standardized effects among all variables in the first model.

#### 4.2.2 Sensitivity Analyses

##### 4.2.2.1 Unconstrained vs. Constrained Models

Next, the full serial mediation model was compared to a model in which all paths but the path from rumination to prolonged grief symptoms were constrained to zero and a model in which paths from and to hopelessness were constrained to zero. The comparison between the fully constrained and unconstrained models showed that the constraints significantly worsened the model fit (𝜒^2^(5) = 148.60, *p* < 0.001). Similarly, the comparison between the partially constrained and unconstrained models showed that the constraints significantly worsened the model fit (𝜒^2^(3) = 77.68 *p* < 0.001). Although we gained three or five degrees of freedom by constraining the paths, the more complex model (i.e., the serial mediation model) fitted the data better in both instances.

##### 4.2.2.2 Reverse Serial Mediation Model

The model fit of the reverse model (i.e., prolonged grief symptoms as independent variable, rumination as dependent variable) was poor (𝜒^2^ = 297.627, *df* = 22, *p* < 0.001; *RMSEA* = 0.088, *CI* [0.054; 0.123]; *CFI* = 0.920; *SRMR* = 0.114). The model explained 65% of the variance in rumination (*R* ^2^ = 0.65) which is a large effect (Kline 2016).

**FIGURE 3:**
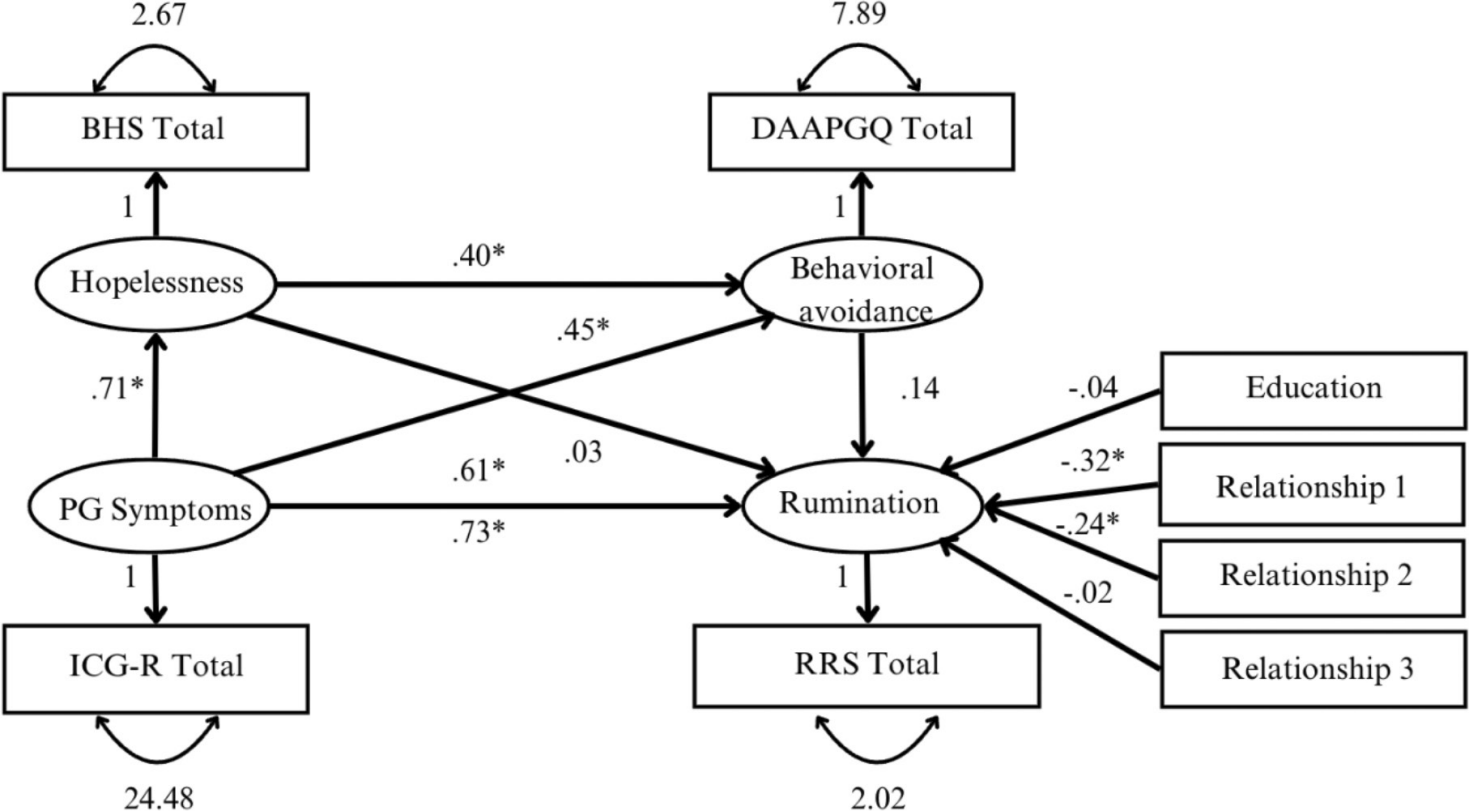
Reverse mediation model including prolonged grief symptoms as predictor of rumination. *Note:* Standardized regression coefficients are depicted. For clarity, the variances of the covariates and the residual errors were omitted. BHS Total = Beck's Hopelessness Scale; RRS Total = brooding subscale of the Ruminative Response Scale; DAAPGQ Total = depressive avoidance subscale of the Depressive and Anxious Avoidance of Prolonged Grief Questionnaire; ICG‐R Total = Inventory of Complicated Grief Revised; PG = prolonged grief. The following variables were dummy‐coded: Education = lower education (0) vs. higher education (1); Relationship 1 = partner (1) vs. parent (0); Relationship 2 = child (1) vs. parent (0); Kinship 3 = sibling (1) vs. parent (0).

The direct effect (c‐path; other paths were fixed to zero) of prolonged grief symptoms on rumination including covariates was significant (*β* = 0.73, *p* < 0.001) indicating that prolonged grief symptoms have a strong positive effect on rumination. Once all other theorized pathways were estimated, the direct effect (c′‐path) became slightly smaller and remained significant, *β* = 0.61, *p* < 0.001. Yet, there were no significant mediation effects. The indirect effect of prolonged grief symptoms via hopelessness on rumination (*β* = 0.06, *p* = 0.199), of prolonged grief via behavioural avoidance on rumination (*β* = 0.02, *p* = 0.860) and of prolonged grief symptoms via hopelessness and behavioural avoidance on rumination (*β* = 0.04, *p* = 0.398) were all non‐significant. Figure 3 illustrates the standardized effects among all variables in the reverse model.

#### 4.2.3 Full Serial Mediation Model Predicting Depression Symptoms

The serial mediation model predicting depression symptoms showed acceptable model fit (𝜒^2^ = 315.035, *df* = 22, *p* < 0.001; RMSEA = 0.087, CI [0.053; 0.122]; CFI = 0.927; SRMR = 0.087). The model explained 79% of variance in depression symptoms (*R* ^2^ = 0.79).

The direct effect (c‐path; other paths are fixed to zero) of rumination on depression symptoms including covariates was significant (*β* = 0.57, *p* < 0.001) indicating that rumination had a strong positive effect on depression symptoms. Once all other theorized pathways were estimated, the direct effect (c′‐path) became smaller (i.e., small effect) and non‐significant, *β* = 0.13, *p* = 0.104.

**FIGURE 4:**
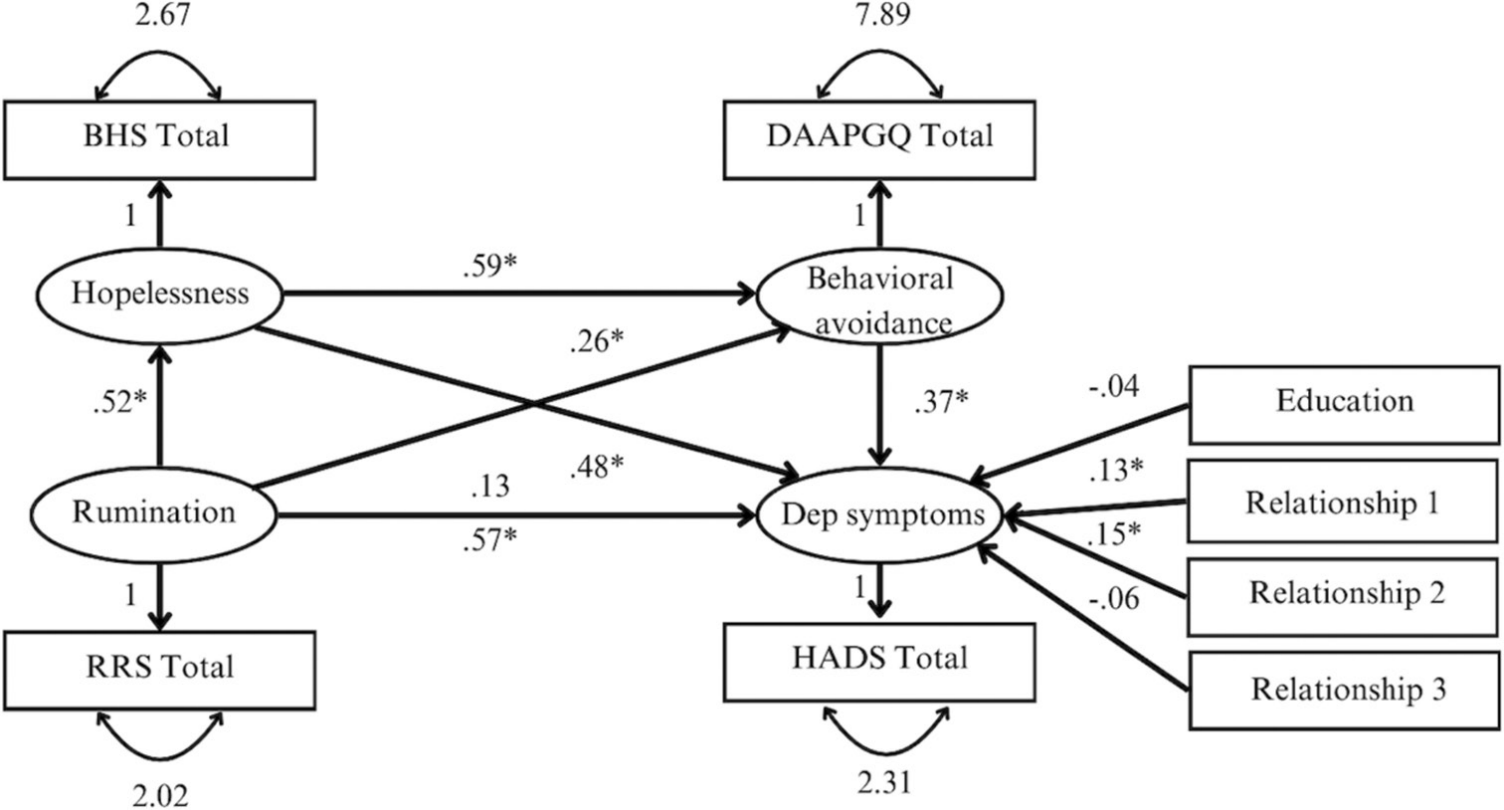
Full serial mediation model predicting depression symptoms. *Note:* Standardized regression coefficients are depicted. For clarity, the variances of the covariates and the residual errors were omitted. BHS Total = Beck's Hopelessness Scale; RRS Total = brooding subscale of the Ruminative Response Scale; DAAPGQ Total = depressive avoidance subscale of the Depressive and Anxious Avoidance of Prolonged Grief Questionnaire; HADS Total = depression subscale of the Hospital Anxiety and Depression Scale; Dep = depression. The following variables were dummy‐coded: Education = lower education (0) vs. higher education (1); Relationship 1 = partner (1) vs. parent (0); Relationship 2 = child (1) vs. parent (0); Kinship 3 = sibling (1) vs. parent (0).

The indirect effects of rumination via hopelessness on depression symptoms (*β* = 0.25, *p* < 0.001), of rumination via behavioural avoidance on depression symptoms (*β* = 0.09, *p* = 0.029) and of rumination via hopelessness and behavioural avoidance on depression symptoms (*β* = 0.11, *p* = 0.004) were small, positive and statistically significant. The contrasts between the indirect effect via hopelessness and the indirect effect via behavioural avoidance (*β* = 0.16, *p* = 0.081), between the indirect effect via hopelessness and the indirect effect via hopelessness and behavioural avoidance (*β* = 0.14, *p* = 0.093), and between the indirect effect via behavioural avoidance and the indirect effect via hopelessness and behavioural avoidance (*β* = −0.02, *p* = 0.694) were not significant. These findings indicate that there are no significant differences between the indirect effects. Figure 4 illustrates the standardized effects among all variables in the model.

#### 4.2.4 Sensitivity Analyses

##### 4.2.4.1 Constrained vs. Unconstrained Models

Next, the model fit of the full serial mediation model was compared to a model in which paths from and to hopelessness were constrained to zero and a model in which all paths but the path from rumination to depression symptoms were constrained to zero. The comparison between the fully constrained and unconstrained models showed that the constraints significantly worsened the model fit (𝜒^2^(5) = 184.75, *p* < 0.001). Similarly, the partially constrained and unconstrained model showed that the constraints significantly worsened the model fit (𝜒^2^(3) = 92.29 *p* < 0.001). Although we gained three or five degrees of freedom by constraining the paths, the more complex model (i.e., the serial mediation model) fitted the data better in both instances.

##### 4.2.4.2 Reverse Serial Mediation Model

The model fit of the reverse model (i.e., depression symptoms as the independent variable, rumination as the dependent variable) was poor (𝜒^2^ = 315.04, *df* = 22, *p* < 0.001; RMSEA = 0.081, CI [0.047; 0.117]; CFI = 0.935; SRMR = 0.106). The model explained 52% of the variance in rumination (*R* ^2^ = 0.52).

**FIGURE 5:**
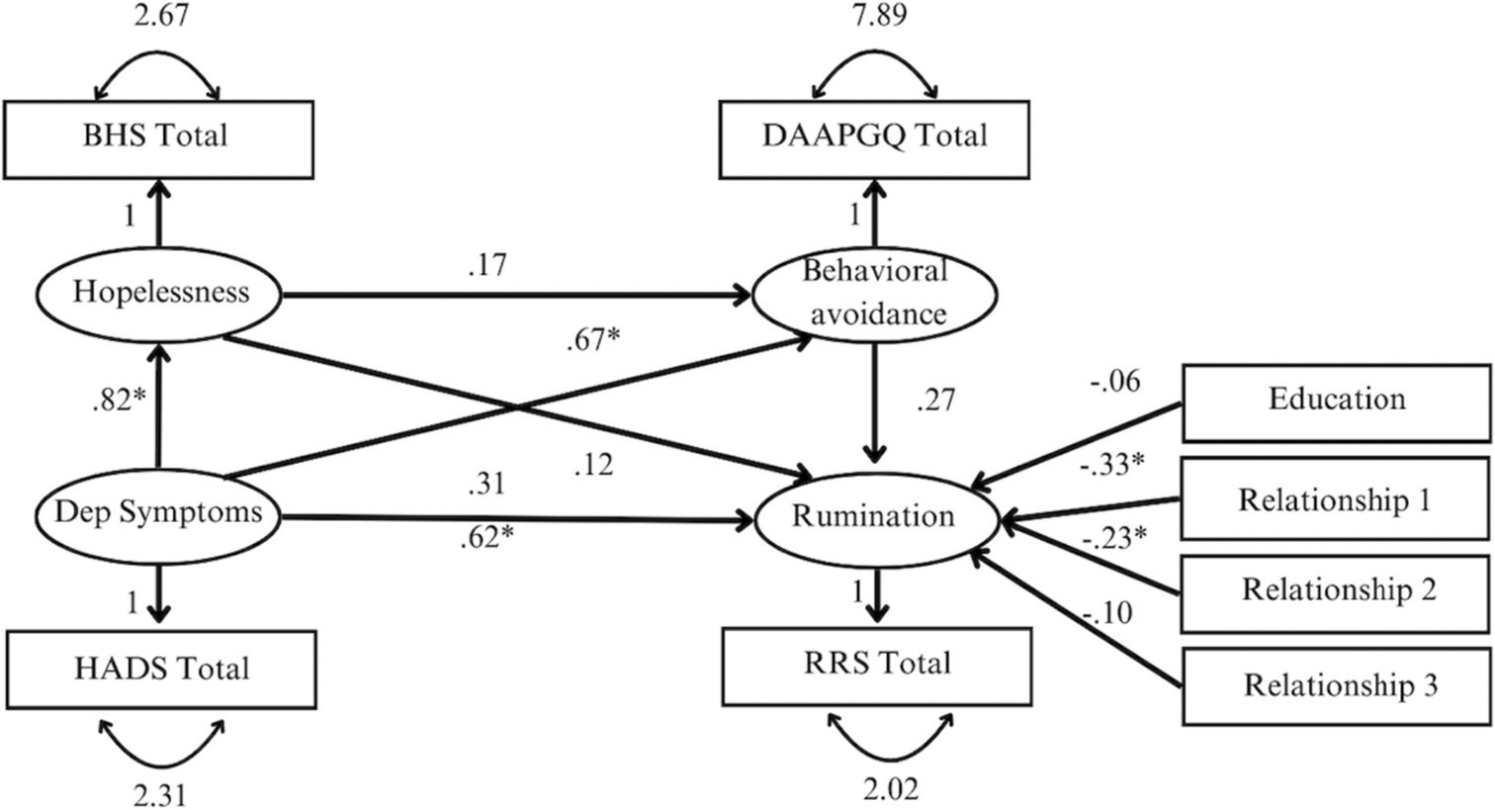
Reverse mediation model including depression symptoms as a predictor of rumination. *Note:* Standardized regression coefficients are depicted. For clarity, the variances of the covariates and the residual errors were omitted. BHS Total = Beck's Hopelessness Scale; RRS Total = brooding subscale of the Ruminative Response Scale; DAAPGQ Total = depressive avoidance subscale of the Depressive and Anxious Avoidance of Prolonged Grief Questionnaire; HADS Total = depression subscale of the Hospital Anxiety and Depression Scale; Dep = depression. The following variables were dummy‐coded: Education = lower education (0) vs. higher education (1); Relationship 1 = partner (1) vs. parent (0); Relationship 2 = child (1) vs. parent (0); Kinship 3 = sibling (1) vs. parent (0).

The direct effect (c‐path; other paths are fixed to zero) of depression symptoms on rumination including covariates was significant (*β* = 0.62, *p* < 0.001) indicating that depression symptoms have a large positive effect on rumination. Once all other theorized pathways were estimated, the direct effect (c′‐path) became smaller (i.e., moderate effect) and was no longer significant, *β* = 0.31, *p* = 0.163. However, no significant mediation effects emerged. The indirect effect of depression symptoms via hopelessness on rumination (*β* = 0.18, *p* = 0.064), of depression symptoms via behavioural avoidance on rumination (*β* = 0.10, *p* = 0.504) and of depression symptoms via hopelessness and behavioural avoidance on rumination (*β* = 0.04, *p* = 0.569) were all non‐significant. Figure 5 illustrates the standardized effects among all variables in the reverse model.

## 5 Discussion

The aim of the present study was to clarify the associations between rumination, hopelessness, behavioural avoidance and depressive and prolonged grief symptoms in a bereaved adult sample. A first serial mediation analysis showed that the relationship between rumination and prolonged grief symptoms was mediated by hopelessness and behavioural avoidance. A second serial mediation analysis demonstrated that the relationship between rumination and depressive symptoms was mediated by hopelessness and behavioural avoidance. Across both models, there were serial mediation effects as well as simple mediation effects of rumination via hopelessness on symptomatology and of rumination via behavioural avoidance on symptomatology. Therefore, there appear to be multiple possible pathways through which rumination may contribute to post‐loss mental health problems.

Theoretically, findings align with the behavioural avoidance mechanism of the RST (Nolen‐Hoeksema, Wisco, and Lyubomirsky 2008). Specifically, it corresponds with the theory that ruminative coping may strengthen perceptions that one's current situation is hopelessly uncontrollable and that one might as well give up. This, in turn, could lead to patterns of inactivity and social withdrawal that could strengthen negative affect and cognitions, thereby perpetuating mental health problems. Additionally, the significant simple mediation by behavioural avoidance in the relationship between rumination and post‐loss depression and prolonged grief symptoms found in both serial mediation models, our findings support the notion that rumination may also directly affect behavioural avoidance by taking up attention and time (Nolen‐Hoeksema, Wisco, and Lyubomirsky 2008). Findings extend earlier findings from a cross‐sectional study showing that behavioural avoidance is a mediator of the association between rumination and prolonged grief and depression symptoms following bereavement (Eisma, de Lang, and Boelen 2020), and those from a longitudinal study demonstrating the same mediating effect in the relationship between rumination and bereavement‐related depression symptoms (Eisma et al. 2013).

An unexpected finding was that another direct mediator of the relationship between rumination and depressive and prolonged grief symptomatology was hopelessness. It makes sense, nevertheless, that perceptions of hopelessness elicited by rumination may also directly negatively affect post‐loss mental health. For example, the feeling that one's situation is hopeless and uncontrollable may elicit feelings of yearning for the lost loved one, a core symptom of prolonged grief (American Psychiatric Association 2022; World Health Organization 2019).

A more elaborate mechanism may also be at work. Following the hopelessness theory of depression, those who are more likely to attribute negative life events to internal, stable and global causes are more likely to become depressed (Abramson, Metalsky, and Alloy 1989; Liu et al. 2015). Following bereavement, ruminators' negative self‐focused thinking style may make it more likely that they perceive a loss to be the result of one's own negative traits (internal), which they think will never change (stable) and will negatively influence all events they might encounter in the future (global), which could elicit depressive episodes characterized by hopelessness. In line with such notions, rumination is associated with grief‐related global negative beliefs about the self, the world and the future, for example, I am worthless, the future is hopeless, as well as negative cognitions on self‐blame, which can exacerbate prolonged grief and depressive symptoms (Boelen and Lensvelt‐Mulders 2005; Boelen, van den Bout, and van den Hout 2006; Doering et al. 2021). Moreover, positive associations have been reported between rumination and negative attributional styles in nonbereaved samples (e.g., Barnum, Woody, and Gibb 2013; Rifkin et al. 2021). Research indicates that rumination is not necessarily maladaptive because of its prolonged focus on negative affect, but rather because of its prolonged focus on negative judgements about the self (Rude, Little Maestas, and Neff 2007). Although not directly tested in the present investigation, prior research further suggests that rumination may be most likely to lead to hopelessness depression and depressive episodes for those with negative inferential styles who tend to attribute negative events to internal and negative traits of the self (Robinson and Alloy 2003). Further research appears indicated to clarify the relative importance and interactions of rumination, attributional styles, negative beliefs and hopelessness in post‐loss mental health.

Another notable finding was that hopelessness and behavioural avoidance explained more of the relationship between rumination and prolonged grief symptoms than of the relationship between rumination and depressive symptoms (i.e., the latter effect was still significant in the full serial mediation model). One interpretation could be that the other proposed mechanisms of the RST (Nolen‐Hoeksema et al. 2008), that is, increased accessibility of negative cognitions, ineffective problem‐solving behaviour, impaired instrumental behaviour and reduced social support, play a role in explaining the remaining direct relationship between rumination and prolonged grief symptoms, but not depressive symptoms. Another interpretation could be that the strong associations between hopelessness, behavioural avoidance and depressive symptoms in this cross‐sectional model did not leave much variance in depressive symptoms to be explained by a direct effect of rumination.

Sensitivity analyses demonstrated that a model not including hopelessness and a model without both mediators did not fit the data better than the full serial mediation analyses. Moreover, reverse mediation analyses did not show significant mediation effects and did not provide a good fit to the data. Together, these findings support the validity of the current serial mediation models.

An additional relevant finding was the strong positive association between prolonged grief and depressive symptoms. These findings complement research showing that symptoms from these related conditions are often strongly related and can reciprocally predict each other (for reviews: Komischke‐Konnerup et al. 2021; Janshen and Eisma 2024). Despite these strong interrelations, factor analytic and latent class analysis studies generally demonstrate that prolonged grief and depressive symptomatology can be distinguished (e.g., Boelen and van den Bout 2005; Dillen, Fontaine, and Verhofstadt‐Denève 2009; Heeke et al. 2023).

From a clinical perspective, our main findings provide further empirical support for theories underlying specific therapeutic strategies for people experiencing severe rumination, prolonged grief and depressive symptoms. Most notably, findings align with the demonstrated effectiveness of behavioural activation, which stimulates bereaved people to increase the number of valued and rewarding activities and can thereby help bereaved people reengage with life without the deceased, which could help lift mood and decrease ruminative coping and post‐loss psychopathology (e.g., Eisma, Boelen, et al. 2015; Papa et al. 2013). Additionally, the results from the present study tentatively suggest that interventions stimulating alternative coping strategies, for example, through problem‐solving skills training (Townsend et al. 2001), might help reduce hopelessness as well as the use of maladaptive coping strategies, such as rumination and social withdrawal, thereby improving post‐loss mental health. Comparing the usefulness of different therapeutic strategies to disrupt the cycle of rumination in bereaved adults in controlled effectiveness studies may prove clinically useful.

## 6 Limitations and Directions for Future Research

Some limitations warrant mention. First, our cross‐sectional correlational design precludes conclusions about causality in the investigated relationships. Although our mediation analyses suggest that hopelessness and behavioural avoidance are working mechanisms in the relationship between rumination and mental health, it is possible that causation could work in the reverse direction to what we theorized. For example, a recent multiwave longitudinal study suggested that depressive and prolonged grief symptoms predict changes in depressive rumination instead of the other way around (Eisma et al. 2022). As another example, relationships between rumination and behavioural avoidance may also be reciprocal, as suggested by longitudinal research showing that rumination predicts behavioural avoidance (Eisma et al. 2013) and randomized controlled trials showing that behavioural activation reduces rumination (e.g., McIndoo et al. 2016; Moshier and Otto 2017). Future longitudinal research may help elucidate the temporal direction of the reported associations in the current study. Second, we could only consider a limited number of main and control variables in the present investigation. We cannot rule out that unobserved third variables (e.g., neuroticism and negative cognitions) may partly explain some of the observed associations.

Third, the sample consisted of predominantly higher educated Western women who lost a spouse or a parent, which could pose a threat to the generalizability of these findings. Replication of this study in a bereaved sample with more lower educated people, men and people with diverse cultural backgrounds is warranted. Fourth, we used the ICG‐R to assess prolonged grief symptoms in this sample, which measures many but not all symptoms of current criteria sets for PGD in the DSM‐5‐TR and ICD‐11 and precludes the establishment of clinical diagnoses (Treml et al. 2020; Stroebe, Schut, and Eisma 2024). Future research on this topic should include validated questionnaires or clinical interviews to assess prolonged grief symptomatology in line with current conceptualizations of PGD, such as the Aarhus Prolonged Grief Disorder Scale (O'Connor et al. 2023) or the Traumatic Grief Inventory Self‐Report Plus (Lenferink et al. 2022). Fifth, we assessed rumination with the brooding subscale of the RRS, which has shown more limited predictive validity than scales specifically designed to assess grief‐related rumination about the causes and consequences of the loss (Eisma and Stroebe 2017, 2021). Findings may have been different had we used a grief‐specific measure of rumination, such as the Utrecht Grief Rumination Scale (Eisma et al. 2014) or the Oxford Grief Coping Strategies Scale (Smith, Wild, and Ehlers 2024).

## 7 Conclusions

The present study demonstrated the complex interplay between rumination, hopelessness and behavioural avoidance in relation to post‐loss mental health problems. Results of the study generally correspond with the theoretical notion that rumination may take up time and serves to build a case that one is facing a hopelessly uncontrollable situation that is difficult to change, thereby contributing to reduced participation in social, recreational and work‐related activities, which could worsen post‐loss mental health. Future longitudinal research should help further disentangle the temporal direction of the reported associations. Taken together with prior survey research, as well as clinical intervention studies, our results suggest that psychotherapeutic interventions focused on increased engagement in meaningful activities that reduce behavioural avoidance and increasing problem‐solving skills that could counteract feelings of hopelessness would be helpful to severely distressed bereaved people showing high levels of rumination.

## Ethics Statement

The Psychology Ethical Committee of Utrecht University approved the current study.

## Consent

All participants provided informed consent.

## Conflicts of Interest

The authors declare no conflicts of interest.

## Data Availability

Data, syntax and output can be obtained from the main author of this paper upon reasonable request.
